# Effect of novel metabotropic glutamate receptor 5 antagonist on gastroesophageal reflux in dogs during anaesthesia

**DOI:** 10.1111/jsap.70053

**Published:** 2025-11-18

**Authors:** A. Glovéus, C. Ekstrand, A. Flöhr, S. Viskjer, O. Höglund

**Affiliations:** ^1^ Evidensia Strömsholm Referral Small Animal Hospital Strömsholm Sweden; ^2^ Division of Food Safety, Infection Biology, Department of Animal Biosciences Swedish University of Agricultural Sciences Uppsala Sweden; ^3^ Department of Biosystems and Technology Swedish University of Agricultural Sciences Alnarp Sweden; ^4^ Division of Small Animal Surgery, Department of Clinical Sciences Swedish University of Agricultural Sciences Uppsala Sweden

## Abstract

**Objectives:**

Gastroesophageal reflux during anaesthesia is a common event occurring in dogs with the potential to cause oesophageal injury and aspiration pneumonia. The objective of the current study was to evaluate the effect of the novel metabotropic glutamate receptor 5 antagonist TT001 on gastroesophageal reflux during anaesthesia in dogs using two different protocols.

**Materials and Methods:**

One hundred and nineteen client‐owned dogs were included and randomly assigned to receive test‐drug containing either active TT001 (*n* = 58) or placebo vehicle (*n* = 61). In protocol 1 (*n* = 77), test‐drug was administered shortly before induction and in protocol 2 (*n* = 42) test‐drug was administered 15 minutes before standardised pre‐anaesthetic drugs. Gastroesophageal reflux (pH <4) was measured and recorded using oesophageal pH‐metry throughout the duration of the anaesthesia.

**Results:**

Gastroesophageal reflux was registered in 45.4% of dogs, in protocol 1: 44.2%, and in protocol 2: 47.6%. No overall statistically significant difference between the dogs receiving active TT001 (group A, 48.3%) and placebo (group B, 42.6%) was observed, regardless of the protocol tested (protocol 1: group A: 47.4%, group B: 41.0%; protocol 2: group A: 50.0%, group B: 45.5%).

**Clinical Significance:**

Administration of metabotropic glutamate receptor 5 antagonist TT001 as a single intravenous bolus injection before or after pre‐anaesthetic drugs has limited effect on the incidence of gastroesophageal reflux in anaesthetised dogs.

## INTRODUCTION

Intraoperative gastroesophageal reflux (GER) is a frequent complication during general anaesthesia that may lead to esophagitis, oesophageal strictures, regurgitation and potentially cause aspiration pneumonia (Bissett et al., 2009; Engelhardt & Webster, 1999; Kook, 2021; Rodríguez‐Alarcón et al., 2015). The prevalence of GER varies substantially between different studies, from as low as 5% (Savvas et al., 2016) to as high as 87.5% (Lambertini et al., 2020), with a large number of studies reporting an incidence of approximately 40 to 60% when using a combination of an opioid and a sedative before elective non‐abdominal surgery (Wilson et al., 2005, 2006, 2007; Viskjer & Sjöström, 2017; Costa et al., 2021; Flouraki et al., 2022). Variations are influenced by differences in methods of measuring and diagnosing GER as well as multiple contributing risk factors including but not limited to, duration of preoperative fasting, pre‐ and anaesthetic drug selection and protocol, age, weight, length of anaesthesia, surgical intervention, and positioning or repositioning during anaesthesia (Anagnostou et al., 2017; Benzimra et al., 2019; Galatos & Raptopoulos, 1995b; Jones & Fransson, 2019; Savvas et al., 2016; Torrente et al., 2017; Tsompanidou et al., 2021; Wilson et al., 2005).

As a result of increased gastric pressure and acid build‐up, mechano‐ and chemoreceptors in the gastric wall become activated resulting in transient lower oesophageal sphincter relaxations (TLESRs) through a reflex pathway leading to the release of built‐up gastric pressure and maintenance of barrier pressure (Franzi et al., 1990; Mittal et al., 1995; Penagini et al., 2004; Stakeberg & Lehmann, 1999; Ullal et al., 2022) which is one of the major factors influencing the incidence of GER (Laitinen et al., 1978; Pratschke et al., 2001; Strombeck & Harrold, 1985). A TLESR is a normal reflex in both man and dogs that leads to a short relaxation of the lower oesophageal sphincter and it is believed that an important cause of reflux episodes in the gastroesophageal reflux disease (GERD) complex is the increase in the number of TLESRs (Dodds et al., 1982; Mittal et al., 1995; Stakeberg & Lehmann, 1999).

Different receptors have been identified in gastric vagal afferents, which are major initiators of TLESRs and may function as interesting therapeutic targets preventing GER (Boeckxstaens et al., 2010; Lehmann et al., 2000; Page et al., 2005). These receptors are primarily metabotropic glutamate receptors (mGluR), and they have been divided into several different groups, and it appears that group 1 and particularly receptor 5 (mGluR5) is strongly associated with reflux (Frisby et al., 2005; Keywood et al., 2009; Rouzade‐Dominguez et al., 2017). Previous studies have evaluated the effect of mGluR5 antagonists on TLESRs in conscious man, ferret and dog with promising results (Frisby et al., 2005; Jensen et al., 2005; Page et al., 2005). TT001 (previously named AZD2066) is a selective non‐competitive antagonist of mGluR5 that was developed primarily for the treatment of GERD and more specifically as an add‐on in refractory GERD cases when the routine treatment regime has failed. The preclinical results are favourable with limited adverse effects, making it suitable for continued research with the current indication (Rohof et al., 2012).

However, cited studies have not evaluated the effect on GER after a single intravenous (iv) bolus injection during general anaesthesia. Therefore, the objective of the current clinical trial was to evaluate the effect of the novel mGluR5 antagonist TT001 on GER in anaesthetised dogs using two different test‐drug administration protocols and the hypothesis was that this novel drug would result in a significant reduction of GER.

## MATERIALS AND METHODS

### Test‐drug administration

The drug TT001 was provided by Orphelion AB (Stockholm, Sweden) in 20 mL vials. A total of 130 vials were delivered blinded, randomised and numbered consecutively from 1 to 130. Half of the numbered vials contained active TT001 (group A), and the other half contained placebo vehicle (group B). The concentration in the vials was 2 mg/mL, and the administered dosage was 0.4 mg/kg. The drug was administered as a slow iv bolus at a maximum rate of 1 mL/min.

### Study design and inclusion criteria

The current study was conducted as a prospective double‐blinded randomised and placebo‐controlled single‐site clinical trial carried out between October 2023 and January 2025 and was approved by the Ethics Committee of the Swedish National Board of Agriculture in Uppsala (licence no. 5.2.18‐07289/2023). All owners were prior to inclusion informed of the intent of the study and a client consent form was signed.

The number of dogs was pre‐determined through a power analysis by the z‐ and Fisher test to reach a power equal to or more than 80% (*α*= 0.05) using a simulation approach. The simulation was based on a balanced design. For group sizes (*n*) between 10 and 150, random draws were made from two binomial distributions, where the assumed probabilities of reflux were 0.5 and 0.25 for treatment A and B, respectively. Two groups of 65 dogs per group were required to reach statistical goals.

A total of 130 client‐owned dogs scheduled for elective orthopaedic surgery were planned. Inclusion criteria were dogs that after clinical examination were deemed clinically healthy (American Society of Anesthesiologists grade 1 to 2), of all ages with a body weight between 2 and 60 kg. Protocol criteria excluded dogs with a history of gastrointestinal disease or diarrhoea/vomiting in the past 14 days, dogs of specific brachycephalic breeds (Boston terrier, English and French bulldog, and pugs), dogs that had received medication that could influence the gastrointestinal tract motility or the mucosal barrier, dogs not fit for the predetermined peri‐anaesthetic protocol due to comorbidities, if the protocol was not followed and dogs that visually vomited or regurgitated after receiving pre‐anaesthetic drugs prior to anaesthesia.

Two different protocols were designed to evaluate the optimal timing of TT001 in a clinical setting. The difference between the two protocols was the timing of test‐drug administration, pre‐ or post‐administration of pre‐anaesthetic drugs.

Owners were instructed to fast their dogs overnight prior to hospital admission. Water was allowed *ad libitum* up until admission. All dogs were administered the pre‐anaesthetic drugs (same for both P1 and P2) consisting of dexmedetomidine (Dexdomitor 0.5 mg/mL, Orion Pharma AB Animal Health, Solna, Sweden; 75 μg/m^2^; body surface area (m^2^) = *K* × (body weight in kilograms)^2/3^, where *K* being a constant for dogs; in the current study, *K* = 0.101) (Allerton, 2020); methadone (Semfortan Vet 10 mg/mL, Dechra Veterinary Products AB, Sollentuna, Sweden; 0.3 mg/kg) given intramuscularly; and, if not already under treatment with an NSAID, an injection of subcutaneous robenacoxib (Onsior 20 mg/mL, Elanco, Solna, Sweden; 1 mg/kg) or iv meloxicam (Metacam 5 mg/mL, Boehringer Ingelheim Animal Health, Uppsala, Sweden; 0.2 mg/kg). In P1, when dogs were appropriately sedated, an iv catheter was placed in a cephalic or saphenous vein and test‐drug was administered accordingly shortly before induction. In P2, the iv catheter had already been placed upon admission in a cephalic or saphenous vein, and the test‐drug was administered iv, 15 minutes before the administration of pre‐anaesthetic drugs. Anaesthesia was induced using propofol (Propovet 10 mg/mL, Zoetis Animal Health ApS, Stockholm, Sweden) until adequate anaesthetic depth for safe orotracheal intubation using a premeasured (measured externally from mandibular incisors to manubrium sterni) endotracheal tube while dogs were positioned in right or left lateral recumbency.

General anaesthesia was maintained using isoflurane (IsoFlo vet 100%. Zoetis Animal Health ApS, Stockholm, Sweden) in 50:50 oxygen and medical air; the flow rate was individualised and adapted to maintain an appropriate surgical anaesthetic depth. Regardless of dog size, a circle re‐breathing system was used, and mechanical ventilation was used if necessary for the dogs’ needs. Dogs scheduled for hind‐limb surgery received an epidural analgesic injection. If CSF filled the needle hub either the needle was redirected until no CSF was retrieved or 1/3 to 1/2 of the total volume was administered. Once the surgical area was clipped and aseptically scrubbed using chlorhexidine soap, the dog was moved into the surgical theatre, placed in the appropriate position for the planned surgical procedure and swabbed with chlorhexidine solution. A single‐use 1.6‐mm pH sensor‐tipped catheter (Digitrapper VersaFlex‐Z. SynMed Medicinteknik AB, Stockholm, Sweden) was calibrated in 7 and 4 pH buffer solutions to ensure correct measurements and then placed in the oesophagus. The measuring tip of the catheter was positioned at the cranial edge of the 10th rib and controlled using radiography or fluoroscopy. Once the correct position was confirmed, the catheter was fixed to the endotracheal tube using adhesive tape and recording was initiated. Readings were made automatically throughout the procedure every minute. Isotonic crystalloid fluid (Ringer‐acetate) was infused at 5 mL/kg/hour continuously throughout the anaesthesia.

### Monitoring and anaesthetic interventions

From the time of admission to general anaesthesia, patients were continuously monitored for vomiting or regurgitation. Once dogs were sedated or anaesthetised, every change or correction of position was denoted in the surgical documentation. Dog handling was careful and kept to a minimum. During general anaesthesia, all dogs were monitored using pulse oximetry, spirometry, non‐invasive blood pressure (NIBP, oscillometry, cuff placed distal antebrachium or metatarsus), 3‐lead electrocardiography, capnography and body temperature (rectally as well as continuously via an intra‐oesophageal probe placed in the cervical oesophagus). Parameters were recorded on average every 5 minutes throughout the anaesthesia.

In case an abrupt increase in heart rate, NIBP or respiratory rate due to surgical stress/pain, a single dose of 3 μg/kg fentanyl was administered iv. If inadequate response and signs of pain were persistent, a second dose of 3 μg/kg fentanyl was administered. If still inadequate response, fentanyl was administered as a cri at an initial dose of 5 μg/kg/hr. If the response was still not appropriate, additional analgesia could be administered and was decided intraoperatively by the surgeon in charge.

In case of hypotension (<65 mmHg mean arterial blood pressure), multiple regimes could be used and appropriate intervention was decided intraoperatively by the surgeon in charge. Crystalloid fluid boluses could be administered (5–15 mL/kg), or isoflurane concentration could be reduced. In case of lack of response, severe hypotension or life‐threatening bradycardia, protocol allowed administration of glycopyrrolate (up to 0.01 mg/kg iv) to desired effect without exclusion from the study.

Before anaesthetic recovery and extubating, the oesophagus of animals with registered GER was lavaged and suctioned with 37°C sterile saline solution until only particle‐free macroscopically clear and odourless fluid could be aspirated in order to reduce the risk of leaving low <4 pH refluxate in the oesophagus. When dogs were fully awake, 3 mL of sucralfate oral suspension (200 mg/mL) was administered, and 1 g sucralfate tablets were prescribed 1 hour prior to feeding and 1 hour after other oral medications three times per day for the following 3 days. Owners were informed to notify the authors if vomiting occurred following GER in the subsequent weeks.

### Statistical analysis

*P*‐values were calculated from Fisher’s exact test and a *z*‐test for proportions. For each value of n, the random draw was repeated 1000 times and the proportion of cases with a *P*‐value below .05 was calculated. The sample size (*n*) was chosen so that the probability of a *P*‐value below .05 was 80%.

Reflux occurrence was tested with Fisher’s exact test on the cross‐table of reflux occurrence and treatment. The numeric response variables duration of reflux (pH below 4), time from recording to reflux and lowest pH, were first tested for normality using a Shapiro–Wilk test. To control for possible treatment and protocol differences, normality was tested after standardising the variables by subtracting treatment and protocol means for each observation. The test rejected the null hypothesis of normality for all three variables, and a difference between treatment groups was therefore tested with Wilcoxon rank sum tests (Mann–Whitney *U* test). The effect of position and positional changes on reflux occurrence was tested with Fisher’s exact test. The Fisher test and the Wilcoxon tests were performed for each individual protocol and for the merged case with both protocols. Results were considered significant at *P* < .05.

Statistical analysis was done in R (version 4.4.2) through RStudio (version 2024.12.0 + 467).

## RESULTS

### Dog inclusion and signalment

One of the 130 vials was destroyed prior to administration because of defective preparation. Due to various reasons for not complying with the predetermined protocol criteria, ten dogs were excluded from the study (the surgical procedure was aborted in three dogs, one dog did not receive test‐drug according to protocol and in six dogs regurgitation was identified after administration of pre‐anaesthetic drugs but prior to induction). After the exclusion of the ten above‐mentioned dogs, a total of 119 dogs were included in the study. The mean body weight, age and sex distribution were similar in groups A and B as displayed in Table 1.

**Table 1:** Descriptive data of 119 client‐owned dogs receiving test‐drug TT001 (GrA) or placebo (GrB) in a randomised, controlled double‐blinded clinical trial evaluating the effect on intraoperative gastroesophageal reflux during elective orthopaedic surgery. The two used protocols are displayed as P1 and P2

	P1 (*n* = 77)		P2 (*n* = 42)		All (*n* = 119)
	GrA (*n* = 38)	GrB (*n* = 39)	GrA (*n* = 20)	GrB (*n* = 22)	
Body weight (kg)	22.2 ± 12.9		19.8 ± 13.1		21.4 ± 13.0
	22.2 ± 11.8	22.3 ± 14.1	21.9 ± 11.1	17.9 ± 14.7	
Age (months)	39.8 ± 35.7		54.6 ± 34.9		45.0 ± 36.0
	34.9 ± 30.6	44.6 ± 40.0	54.0 ± 26.9	55.2 ± 41.4	
Males	43		23		66
	21	22	9	14	
Females	34		19		53
	17	17	11	8	

### Gastroesophageal reflux incidences

The overall incidence of GER was 45.4%. The difference between the two protocols was non‐significant (P1: 44.2%, P2: 47.6%, *P* = .87). When evaluating the effect of the test‐drug, separating the dogs into group A and group B, the incidence of GER was 47.4% and 41.0%, respectively, in P1 (*P* = .65) and 50.0% and 45.5% in P2 (*P* = 1.00); the overall incidence of GER was 48.3% in group A and 42.6% in group B (*P* = .58).

### Oesophageal pH‐metry measurements

The period from induction to probe placement represented a blind period when GER could occur without being registered as the dogs were anaesthetised without oesophageal pH‐metry in place. The overall average time between induction and the start of recording was 31.4 minutes (P1 35.0 minutes, P2 24.9 minutes). In 24 cases (24/54, 44%), GER had already occurred when the probe was inserted and started. In the remaining 30 (30/54, 56%) cases, GER occurred in the surgical theatre. Registered GER <4 pH occurred on average 18.9 minutes after the start of recording and there was no statistical difference found comparing group A and group B (21.7 and 15.8 minutes, respectively, *P* = .25). The average duration of the GER episodes was 56.8 minutes. There was no significant difference between group A and group B (57.8 and 55.6 minutes, respectively, *P* = .67) and the overall mean duration of the pH‐metry recording was 103.5 minutes (group A 104.6 minutes, group B 102.4 minutes).

### Contributing factors

The duration of preoperative fasting was known in 96 dogs, and in 23 dogs, it was not specified but considered >8 hours according to instructions given to owners before inclusion. The overall average time of preoperative fasting was 15.6 hours in cases where the duration was known. In the GER group, it was 15.5 hours, and in the nGER group, it was 15.6 hours which was not a significant difference (*P* = 1.00).

Once the study dogs were sedated or anaesthetised enough to be handled or moved safely, positional changes and pressure on the abdomen were kept to a minimum to reduce influence on GER. However, positional changes could not be avoided. During surgical intervention, dogs were positioned in four different positions depending on the intervention: dorsal, sternal, left lateral and right lateral recumbency. The majority of dogs, 66% (*n* = 78/119) were in the dorsal position, 16% (*n* = 19/119) in left lateral recumbency, 14% (*n* = 17/119) in right lateral recumbency and 4% (*n* = 5/119) in the sternal position. No reflux episodes were registered in sternal recumbency, (a non‐significant trend comparing sternal position to dorsal, left lateral and right lateral recumbency, *P* = .063). No statistical significance was found for the remaining positions. Most dogs positioned dorsally were repositioned (59%, *n* = 46/78), of which 37% (*n* = 17/46) registered GER.

## DISCUSSION

In the current clinical trial, the overall incidence of GER in the study population was 45.4%, which is comparable to previous similar studies. No statistical difference in the incidence of GER was demonstrated between study groups group A (48.3%) and group B (42.6%) regardless, of whether TT001 was administered before or after pre‐anaesthetic drugs.

The first protocol (P1) was theoretically the most effective and in a clinical setting also the most practical to carry out. The second protocol (P2) was developed to allow complete priming of the mGluR5 receptors prior to administration of any other drugs to exclude their potential interaction with mGluR5 limiting the efficacy of TT001. However, there are no known direct interactions with the used peri‐anaesthetic drugs and TT001.

In the present trial, manual pressure of the abdomen was minimised peri‐anaesthetically to reduce abdominal pressure peaks that may have predisposed to GER (Galatos & Raptopoulos, 1995a; Garcia, 2013). Despite clear protocol instructions regarding how to move and reposition the patients during anaesthesia, pressure on the abdomen directly or indirectly could not be avoided. In the current study, the time between induction and probe placement was on average 31.4 minutes. Almost half (44%, 24/54) of the dogs in the GER group had refluxed at the start of recording indicating that the reflux occurred sometime between induction and probe placement, a period from which we have no recordings. Our results demonstrated that GER frequently occurred shortly after induction. A finding that is in coherence with available literature, suggesting that dogs often reflux early after induction (Flouraki et al., 2022; Shaver et al., 2016; Tsompanidou et al., 2021). It is also suggested that longer anaesthesia times carry an increased risk of reflux due to acid build‐up and gastric pressure (Benzimra et al., 2019), a finding that was not confirmed in the current study.

Numerous studies have failed to identify an effect of a particular body position on intraoperative GER, and in studies reporting a predisposition to a certain position the results are often inconsistent and inconclusive (Benzimra et al., 2019; Favarato et al., 2011; Flouraki et al., 2022). One study reported an increased risk of GER in sternal recumbency which is similar to the findings of the current trial indicating a potential risk with sternal position (Pratschke et al., 2001). Yet, another reported a potential increased risk of GER in dorsal recumbency, due to a decrease in the lower oesophageal sphincter pressure (LESP) (Waterman et al., 1995). The latter finding may be confirmed in another study that reported an increased incidence of GER in dorsal recumbency, possibly attributed to the aforementioned reduction in LESP (Viskjer & Sjöström, 2017), but this was not observed in the current study. Torrente et al. (2017) found that the change in position was the most important factor increasing the risk of GER (Torrente et al., 2017). A very interesting finding that could be explained by the change in abdominal pressure and reduction in barrier pressure. In the present study, the increased number of positional changes did not increase the risk of GER.

In clinical settings, the authors of previous publications suggested that mGluR5 antagonists may have therapeutic value in the treatment of GERD attributed to the dose‐dependent inhibitory effect on TLESRs (Frisby et al., 2005; Jensen et al., 2005; Zerbib et al., 2011). A similar efficacy was reported in another study evaluating mavoglurant, another mGluR5 antagonist; however, at the effective dosage, numerous clinical adverse effects were reported prohibiting its use as a therapeutic agent (Rouzade‐Dominguez et al., 2017). Moreover, in healthy conscious humans a single oral dose of TT001/AZD2066 caused a significant reduction in the number of TLESRs and reflux episodes of up to 27% and 51%, respectively, without severe adverse effects (Rohof et al., 2012). Summarising the results of both pre‐clinical trials of mGluR5 antagonists in dogs and clinical trials in humans has been promising, demonstrating a dose‐dependent reduction in the number of TLESRs and reflux episodes (Frisby et al., 2005; Jensen et al., 2005; Rohof et al., 2012; Rouzade‐Dominguez et al., 2017; Zerbib et al., 2011). Similarly, when TT001 was administered to dogs as an iv infusion over 60 minutes at a dosage of 0.4 mg/kg, the number of TLESRs was reduced by 49 ± 10% (Orphelion, 2023). Considering the available literature and results reported and the importance of TLESRs in the pathogenesis of GER, we hypothesised that there would be a reduction of GER episodes in the present clinical study using TT001, an mGluR5 antagonist.

The reason why the results of the current study did not demonstrate a reduction in GER after administration of TT001, was not clearly identified. Possible attributing factors were dosage, administration route and others such as movements and repositioning of patients that are difficult to standardise in a clinical setting. The authors speculate whether TT001 is effective as a constant rate infusion (CRI) but not as a bolus when administered to dogs. A previous study using another mGluR5 antagonist in dogs, reported a significant reduction in the number of TLESRs after iv bolus injection, indicating that iv bolus injection may be efficient (Rouzade‐Dominguez et al., 2017). In unpublished data, the terminal half‐life of TT001 was 2.3–4.1 hours after iv administration in dogs suggesting rapid initial and intermediate half‐lives (Orphelion, 2023). In the current study, the time from test‐drug administration to the end of recording was 138 and 172.3 minutes (P1 and P2, respectively). Consequently, plasma concentrations may have fallen below effective concentrations indicating that the effect of the test‐drug may have dissipated throughout the length of the anaesthesia. Under those conditions, a CRI could be beneficial. However, 44% of the GER episodes occurred within 35 and 68minutes (P1 and P2, respectively) which suggests a less significant fall in plasma TT001, and therefore, disputes the above statement further complicating the interpretation of current results.

Available conducted mGluR5 antagonist studies have been carried out on conscious humans or dogs. In the current study, patients were anaesthetised, which according to the authors’ knowledge has not yet been evaluated. mGluR5 antagonists have the primary indication GER, which is a disease in conscious humans and dogs (Muenster et al., 2017) and not anaesthetised patients. In the current study, we were evaluating the effect on intraoperative GER which is a different disease with a different pathophysiology. According to two studies evaluating TLESRs in healthy conscious and sleeping humans, they concluded that TLESRs were only present when the subjects were conscious (Dodds et al., 1982; Freidin et al., 1991). Cox et al. (1988) evaluated the presence of TLESRs during anaesthesia using oesophageal manometry and gastric insufflation in four dogs (all dogs were tested both in conscious and anaesthetised states) and could not see any signs of TLESRs during anaesthesia (Cox et al., 1988). These findings indicate that TLESRs are under CNS control and require consciousness to be functional in both man and dog. Considering the results from the current study as well as the results from the literature reviewed and cited, it appears likely that TT001 and other mGluR5 antagonists cannot reduce the incidence of GER during anaesthesia, due to the absence of TLESRs in an unconscious state.

In the present study, there were several limitations that may have influenced the results and evaluation of TT001. This study was conducted in a clinical setting with true patients, which led to a multivariate setting. Many of the known contributing factors, including surgical intervention, body position, body weight and preoperative fasting were not standardised due to the clinical setting, which may have led to bias in the groups. In the current study, rescue analgesia and glycopyrrolate were allowed without exclusion according to protocol but considering the potential effect on LESP caused by opioids and anticholinergics, maybe patients receiving these drugs should have been excluded regardless of the fact that no direct causality was observed between the drug administration and episodes of GER. Moreover, dogs were moved from one table to another using manpower when stretchers may have provided a better alternative to minimise unnecessary abdominal pressure. Additionally, the pH‐probe was removed prior to oesophageal lavage preventing assurance that the remaining oesophageal fluid was >4 pH.

In conclusion, in the current study, GER during anaesthesia was a common event that occurred in almost half of the patients and frequently had an early onset after induction. Despite promising pre‐clinical results, the study failed to show a reduction in the incidence of GER using TT001 compared to placebo control. Therefore, the study hypothesis was rejected. The current study is the only one of its kind and additional prospective blinded randomised and placebo‐controlled clinical trials are needed using other routes and methods of administration to correctly assess the value of TT001 and other mGluR5 antagonists in the anaesthetised patient.

## Author contributions

A. Glovéus: Planning and organisation of study; organised and performed preoperative evaluation and exams; monitored the measurements and procedures; performed data collection; data curation and formal analysis; drafting and revising the manuscript. C. Ekstrand: Data analysis; drafting and revising the manuscript. A. Flöhr: Performed statistical analysis; data curation and formal analysis; drafting and revising the manuscript. S. Viskjer: Conceptualisation; planning and organisation of study; organised and performed preoperative evaluation and exams; monitored the measurements and procedures; performed data collection; drafting and revising the manuscript. O. Höglund: Conceptualisation; planning and organisation of study, data analysis; drafting and revising the manuscript. All authors approved the final manuscript.

## Conflict of interest

Company Orphelion AB has consulted the author OH regarding the veterinary use of pharmaceuticals. The author OH did not participate in data collection/surgeries; treatment and statistical analysis was performed in a blinded fashion. The other authors have no conflicts of interest to declare.

## Funding

The authors thank Stiftelsen Strömsholm Djursjukvård for financial support to be able to conduct the current clinical trial in a private practice setting and Orphelion AB for providing the prepared test‐drug vials.

## Data Availability

The data that support the findings of this study are available on request from the corresponding author (AG).
